# The Impact of Process Parameters on Microstructure and Mechanical Properties of Stainless Steel/Carbon Steel Clad Rebar

**DOI:** 10.3390/ma12182868

**Published:** 2019-09-05

**Authors:** Ying-ying Feng, Huan Yu, Zong-an Luo, R.D.K. Misra, Guang-ming Xie

**Affiliations:** 1 The State Key Laboratory of Rolling Technology and Automation, Northeastern University, Shenyang 110819, China (Y.-y.F.) (Z.-a.L.) (G.-m.X.); 2 Department of Metallurgical, Materials and Biomedical Engineering, University of Texas at El Paso, El Paso, TX 79968-0521, USA

**Keywords:** stainless-steel clad rebar, deformation degree, deformation temperature, microstructure evolution, mechanical properties

## Abstract

In this study, a 304/20MnSi stainless-steel clad rebar was prepared by single-pass compression process using the MMS-200 Thermal Mechanical Simulator. The impact of different degrees of deformation and deformation temperature on microstructure evolution and the mechanical properties of stainless steel clad rebars were investigated. The study indicated that with the increase of the degree of deformation, the content of pearlite in a carbon steel matrix was increased, and the grains refined. The metallurgical bonding of the bonded interface was formed under high temperature and high extrusion force. With the increase of the deformation temperature, more bainite was obtained on the side of carbon steel, and the grain size increased. The obvious diffusion of Fe, Cr and Ni elements near the bonding interface resulted in higher microhardness of the stainless steel side and smaller microhardness of the carbon steel side. Moreover, the engineering stress-strain curves obtained by the tensile test showed that the plastic deformation of stainless steel and carbon steel was more coordinated. With the increase of deformation temperature and the degree of deformation, the tensile strength of the stainless steel clad rebar was as high as 690 MPa and the elongation was 26%, which was superior to the properties of the clad rebar prepared by other process parameters.

## 1. Introduction

Due to its high service strength and low cost, the traditional carbon steel rebar has been widely used in the field of construction as reinforced concrete skeletons, but huge economic losses are caused easily because of corrosion. The stainless steel rebar, as one of the representative products of the corrosion-resistant rebar, has been gradually applied because of its excellent corrosion resistance, but its high cost limits its extensive use. The stainless steel/carbon steel clad rebar, which has the advantages of both the carbon steel rebar and the stainless steel rebar, has attracted more and more attention in recent years. Thus, it is considered to be one of the most promising new building composite materials [1,2,3].

The stainless steel/carbon steel clad rebar is made by using a stainless steel pipe or strip as cladding material and a carbon steel bar as the core material. At present, there are many research processes being carried out on the stainless steel clad rebar. Sawiki et al. [4,5] prepared X2CrNi18-10/C45E clad rebar by explosion and rolling composite method, and the microstructure and mechanical properties were studied. The results showed that the cladding metal distribution was uneven, and the interface bonding performance was poor. Cross et al. [6] prepared a stainless steel clad rebar by the hot roll-cladding method, and the results of the microstructure, tensile test and impact tests indicated that the combination of core carbon steel and cladding stainless steel was not tight and a brittle fracture occurred during the mechanical test. Mudhaffar et al. [2] studied the microstructure and mechanical properties of the stainless steel clad rebar by designing different passes and selecting different rolling process parameters. The results showed that the bimetal formed strong metallurgical bonding with better bonding performance. It was concluded that the rolling speed compared with rolling temperature, rolling direction and reduction ratio had a smaller effect on the mechanical properties of the stainless steel clad rebar. Gao et al. [7] simulated the rolling process of the stainless steel clad rebar by finite element modeling and the effect of push/tension on the deformation process was studied. Next, a comparative experiment was carried out. The results showed that thrust was beneficial to improving the metallurgical bonding of bimetals, but the process was complicated. Xie et al. [8] penetrated the Q195 carbon steel into the 304 stainless steel seamless pipe, and drew with the wire drawing machine to produce a certain pre-tightening force between the two metals, welded seals at both ends and heating, then the stainless steel clad rebar was prepared by rolling. A closer metallurgical bonding, uniform cladding distribution and better mechanical properties of the stainless steel clad rebar were obtained, but the experimental process was more complex, and the bonding properties fluctuated. Xiang et al. [9] realized the significance of the interference fit cleaned stainless steel pipe and carbon steel core under the action of press, then welded both the ends in vacuum and placed them into the furnace to 1050–1280 °C, followed by cooling to a certain temperature and multi-pass hot rolling. The metallurgical bonding of carbon steel and stainless steel could be achieved by this process under the dual effect of interference fit and rolling, but there were some problems such as unstable bonding performance and uneven thickness distribution of cladding. Thus, stainless steel and carbon steel cannot achieve solid metallurgical bonding, and uneven cladding distribution and low bonding strength, are the key factors that restricted the wide use of the stainless steel clad rebar. Therefore, the key issues of bonding state and bonding performance of the composite interface need to be further studied, and the appropriate methods need to be further explored.

**Figure 1:**
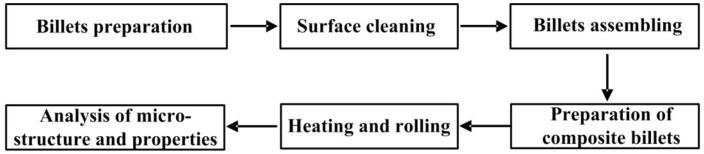
Process route of hot-rolled composite method.

The use of thermo-mechanical techniques to study the microstructure and properties of materials has become an important part. Egea et al. [10] used 308L stainless steel wires as experimental material and the thermo-mechanical model, the numerical simulations and the microstructure analysis of electrically assisted wire drawn specimens were investigated. The results showed that thermo-mechanical methods can evaluate and improve the formability of materials. The thermal simulation technology which is based on the actual production process, simplifies the process conditions, and the prototype is replaced by the experimental model, and an equal proportion of sample or substitute material is used to simulate the production process on the equipment [11]. This method can reveal the mechanical properties and microstructure evolution of metal materials during hot working deformation compared with the “empirical” method. It also provides a good prediction of the problems that may occur during the process of thermal processing, which provides strong technical support and a theoretical basis for streaming the production process and the research and development of new materials. In addition, compared to the laboratory hot rolled-cladding method (the production process is shown in Figure 1), the thermal simulation experiment method has many advantages such as a short experimental period, good experimental repeatability and low experimental cost. Therefore, in this study, the rolling deformation process of the stainless steel clad rebar was simulated by single pass compression process, which can be done on the MMS-200 thermal mechanical simulator in the laboratory. This was self-developed by the State Key Laboratory of Rolling and Automation of Northeastern University. Next, the effects of two different degrees of deformation (50% and 70%) and two different deformation temperatures (950 °C and 1050 °C) on the microstructure and mechanical properties of the bonded interface of stainless steel rebar were studied, which could provide theoretical basis and technical guidance for the preparation of stainless steel clad rebar by hot rolled-cladding method in actual production.

## 2. Materials and Experimental Procedure

**Figure 2:**
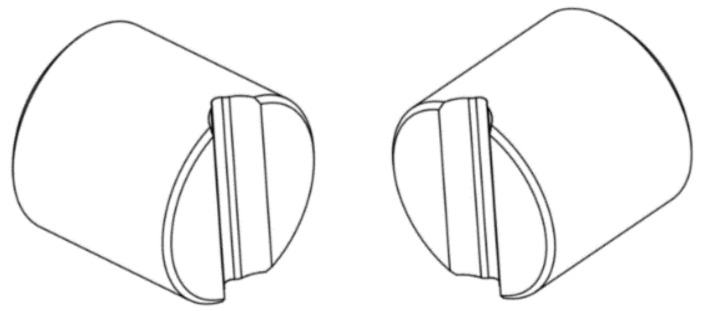
Fixture for thermal simulation experiment.

The core and clad materials of the stainless steel clad rebar were 20MnSi carbon steel and 304 austenitic stainless steel, respectively, and the chemical composition of both these materials are shown in Table 1. The dimensions of the 304 stainless steel and 20MnSi were Ø12 mm × 1.5 mm × 25 mm (outer diameter × wall thickness × length) and Ø9 mm × 25 mm (diameter × length), respectively. Then the MMS-200 thermal mechanical simulator (Northeastern University, Shenyang, China) and the specially designed fixtures (as shown in Figure 2) were used to do single pass compression experiments, and the effects of different process parameters on the microstructure and properties of stainless steel/carbon steel clad rebar were studied.

**Figure 3:**
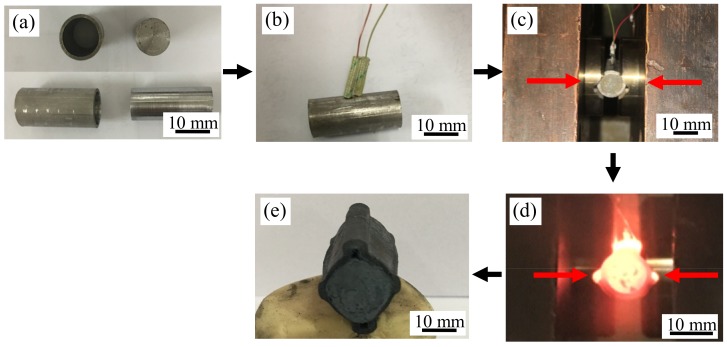
Process steps of preparing stainless steel clad rebar by thermal simulation experiment: (**a**) surface cleaning and blanks assembling; (**b**) welding thermocouple; (**c**) clamping blanks; (**d**) single pass compression experiment; (**e**) stainless steel clad rebar.

A schematic of stainless steel clad rebar which was prepared by the thermal simulation experiment method is shown in Figure 3, it includes four steps, (1) surface cleaning and assembly; (2) welding thermocouple; (3) clamping sample; and (4) single pass compression. The detailed process is as follows: the inner surface of the stainless steel pipe and the outer surface of the carbon steel bar were cleaned first, and after assembly, a pair of thermocouple wires were firmly welded on the center of the composite billet surface by a thermocouple welding machine through the electro-percussive welding method. Then, the composite billet with the thermocouple wire was clamped to the fixtures on both sides of the operation box of MMS-200 thermal mechanical simulator, and a pair of thermocouple wires was connected to the positive and negative poles of the terminal box in the operation box, respectively. Finally, a single pass compression experiment was used.

**Figure 4:**
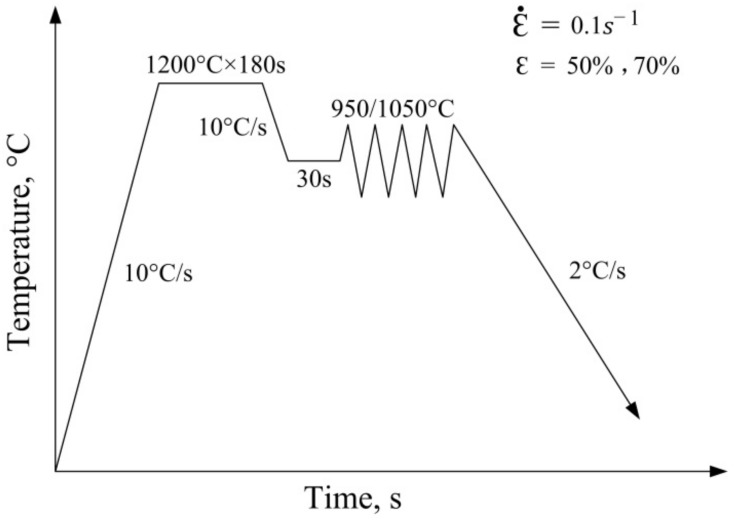
Process route of single pass compression.

The single pass compression process is shown in Figure 4. Firstly, the composite billet is clamped and fixed, then the operation box of the thermal mechanical simulator is vacuum-pumped and filled with protective gas to avoid oxidation of the composite billet. Next, the process of simulated rolling deformation is carried out according to the single pass compression process. Then the composite billet was heated to full austenitized temperature and held for 180 s, and it was compressed at the deformation temperature. Then on-line cooling of composite rebar sample was performed according to the given cooling process. Finally, a stainless steel clad rebar was obtained.

**Figure 5:**
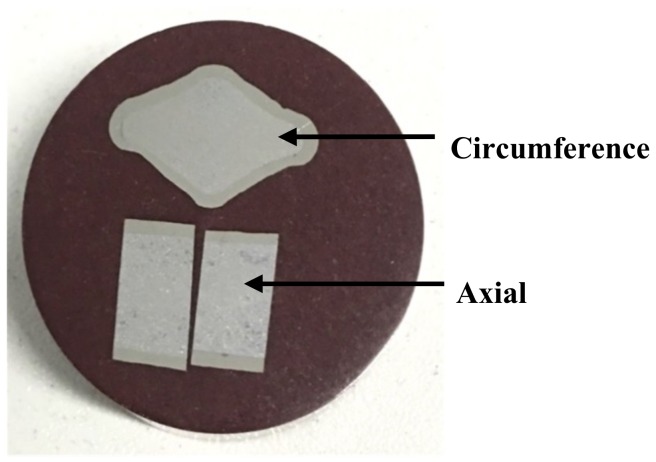
Cross-sectional morphology of stainless steel clad rebar.

Its circumferential and axial cross-sectional morphology are shown in Figure 5. It can be seen that the appearance of the circumferential and axial sections of stainless steel clad rebar is complete, the region of clad rebar rib simulated by a small specimen also shows the expected bulge, the interface is close and continuous, there are no creases, cracks or other defects, and the thickness distribution of stainless steel cladding is more uniform.

In the study described here, the effect of different degrees of deformation and deformation temperatures on microstructure evolution and the mechanical properties of stainless steel/carbon steel clad rebars were studied. First, the same deformation temperature was set at 1050 °C, and the relationship between microstructure evolution and the bonding performance of the stainless steel clad rebar after different degrees of deformation (50% and 70%) at this temperature was studied. Similarly, the same degree of deformation was set at 70%, and the relationship between the microstructure evolution and the bonding performance of the stainless steel clad rebar at different deformation temperatures (950 °C and 1050 °C) was studied.

Metallographic specimens were cut along the axial and circumferential directions near the thermocouple of the stainless steel clad rebar of the above process, respectively. The specimens were mechanically polished and then etched with 4% nitric acid alcohol solution. The interfacial microstructure and compositions were observed using optical microscope (OM, Leica, Wetzlar, Germany), scanning electron microscope (SEM, Oberkochen, Germany) and electron probe microanalysis (EPMA, JEOL, Tokyo, Japan) equipped with wavelength dispersive spectroscopy (WDS, JEOL, Tokyo, Japan). The microhardness distribution of the metal on both sides of the interface was measured by FM-700 microhardness tester (FUTURE-TECH, Tokyo, Japan) at a load of 300 gf for 10 s.

**Figure 6:**
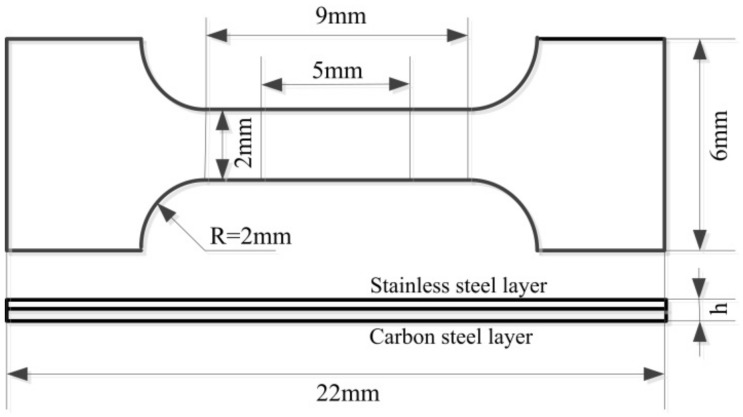
Schematic diagram of the tensile test sample.

The strength, lifetime and properties of laminar metal materials are generally evaluated by measuring the bonding strength of the composite interface. The measurement of composite metal strength is more complex than the single metal strength, especially involving composite wires, bars, etc. There is no uniform method or description to guide such experiments at present. The mechanical properties of the stainless steel clad rebar studied in this paper are difficult to measure due to their small size. In contrast, the tensile test was easier. The detailed process was that a rectangular specimen with length of 25 mm and width of 10 mm was cut from the stainless steel clad rebar of different process by wire cutting machine, then the surface of the specimen was smoothed and a mini non-standard tensile specimen with gauge length of 5 mm, width of 2 mm and thickness of h mm (h was about 2 mm) was cut from the above rectangular specimen. After the photosensitive strip was accurately attached to the tensile specimen, then the mechanical property of stainless steel clad rebar was tested on the WDW-50 tensile testing machine (INSTRON, Boston, MA, USA). The tensile strain was measured by a laser extensometer and the engineering stress-strain curves of the clad rebar made by different process parameters were obtained. The schematic diagram of the mini tensile specimen is shown in Figure 6.

## 3. Results and Discussion

### 3.1. Analysis of the Composite Interface

**Figure 7:**
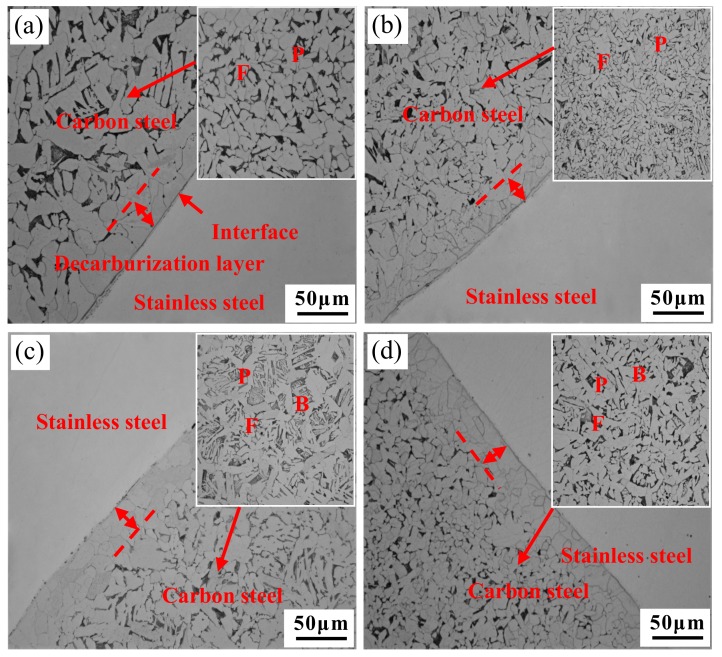
Microstructure of stainless steel clad rebar under different process parameters: (**a**) T = 950 °C, Ɛ = 50%; (**b**) T = 950 °C, Ɛ = 70%; (**c**) T = 1050 °C, Ɛ = 50%; (**d**) T = 1050 °C, Ɛ = 70%.

Figure 7 shows the microstructure of the bonded interface of stainless steel clad rebar for different process parameters. The unetched part of the OM images is the stainless steel side, and the part with many grains is the carbon steel side.

It can be seen from the OM images that the stainless steel/carbon steel clad billet was closely combined with each other after the single-pass compression, and the interface was relatively straight. The composite interface can be divided into four sections: stainless steel matrix, composite interface, decarburization layer and carbon steel matrix. The microstructure of carbon steel matrix was different for different processes. When the clad rebar underwent a small degree of deformation at a lower temperature, that is, T = 950 °C, Ɛ = 50%, ferrite and pearlite were mainly obtained in the carbon steel matrix. Given that 50% degree of plastic deformation was given at 950 °C, the phase transition was more likely to occur, and deformation could promote phase transition of ferrite and a large amount of pro-eutectoid ferrite is expected to precipitate during the cooling process. When temperature was lowered to the pearlite transformation zone, a small amount of retained austenite transformed into pearlite and when temperature continued to cool to bainite transformation zone, there was almost no residual austenite to form bainite. In addition, the lower deformation temperature suppressed bainite transformation, so the room temperature microstructure after transformation was ferrite and pearlite. However, with an increase in the degree of deformation or deformation temperature, the microstructure of carbon steel matrix was ferrite, pearlite and bainite. When deformed at a higher temperature, phase transition was relatively slower and lasted longer, and a small amount of pro-eutectoid ferrite was formed during cooling process. It can be noted from the literature [12] that when a small amount of pro-eutectoid ferrite was precipitated before bainite transformation occurred during cooling process, deformation could promote the phase transformation of bainite. And this could be validated by the Continuous Cooling Transformation curve, which can be simulated by JMatPro software. Moreover, when the degree of deformation was large, the content of the pearlite increased which can be seen from above microstructure (Figure 7d). This was because the degree of destruction of austenitic grains increased with deformation, the grain boundary energy increased and so did diffusion of carbon atoms from crushed grains, so that the pearlite content was increased [13].

The microstructure of the bonded interface of stainless steel clad rebar after 50% degree of compression deformation is shown in Figure 7a,c. It can be seen from the figures that some fine granular inclusions were distributed near stainless steel matrix and a decarburization layer with a thickness of 20–30 μm was formed near carbon steel side of the composite interface. The decarburization layer was formed due to large concentration gradient between carbon steel side and stainless steel side. The mass fraction of C in carbon steel was 0.25%, and the mass fraction in stainless steel was 0.05%. Thus, C diffused into stainless steel side under high temperature and deformation conditions [14]. Figure 7b,d show the microstructure of the bonded interface after 70% degree of compression deformation. It is clearly seen that there was only a small amount of oxidized inclusions near the bonded interface. This was because degree of deformation stainless steel clad rebar continuously increased in the process of compression deformation, and oxides formed near the composite interface during heating process were first wrapped into the matrix under the action of large extrusion force and interface shear force, then gradually crushed [15]. Moreover, the area of the composite interface increased gradually, and inclusions were also dispersed with compression deformation. Therefore, with the increased degree of deformation, the size of inclusions of the composite interface became smaller and smaller, and the number of inclusions per unit area became less and less. At the same time, it can be seen that decarburization layer was also formed near the carbon steel side of the composite interface, but the thickness was reduced to 10–18 μm compared with that in Figure 7a,c. The thickness reduction of decarburized layer was due to the increased degree of deformation, the outer diameter of clad rebar was continuously compressed, the thickness of original carbon diffusion layer also decreased with compression. During the process of plastic deformation and cooling, the diffusion of the composite interface would increase the thickness of the diffusion layer after compression, but the diffusion distance was still smaller than the original carbon element after heating. Figure 7b,d show that thickness of the decarburized layer decreased with the increase of temperature after 70% compression deformation. The reason was that the element diffused rapidly at high temperatures. During hot deformation, temperature is the most important factor which affects the element diffusion rate. The higher the deformation temperature, the more intense the thermal vibration of the atom, and the greater the probability that the atom will be activated to migrate, and more conducive to diffusion. Therefore, with increased deformation temperature, the larger the activation energy of C, the greater is the driving force for movement, and the longer the diffusion distance from carbon steel side to stainless steel side. However, when the temperature increases to a certain value, the C element diffused to the far side of the stainless steel. Therefore, a large concentration gradient was formed near the interface, and C of carbon steel, which was far away from the interface, would migrate to the decarburized layer, which increased the content of C in the decarburized layer and decreased the width of the decarburized layer.

**Figure 8:**
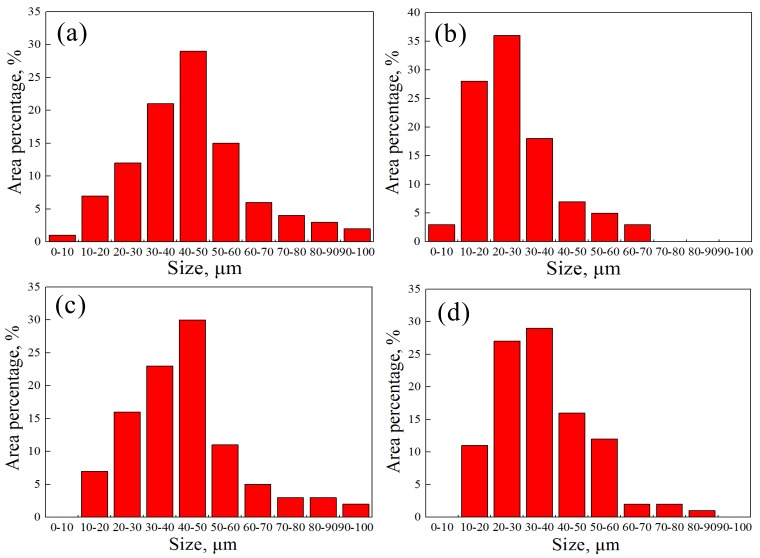
The size distribution of ferrite of carbon steel side under different process parameters: (**a**) T = 950 °C, Ɛ = 50%; (**b**) T = 950 °C, Ɛ = 70%; (**c**) T = 1050 °C, Ɛ = 50%; (**d**) T = 1050 °C, Ɛ = 70%.

From the comparison of grain size of Figure 7a,b and Figure 7c,d, it can be seen that the grain size of Figure 7b is smaller than Figure 7a, and the grain size of Figure 7d is smaller than Figure 7c. The statistics of the grain size of ferrite are shown in Figure 8.

When the degree of deformation was greater, the grains were squeezed to become flat and defects such as dislocations and deformation bands were likely to become nucleation sites, the number of grain boundaries were also increased, and the stored deformation energy was more, which provided a more sufficient driving force for the occurrence of plastic deformation, and finer grains were easily obtained [16,17].

From the above microstructure analysis, it can be seen that the grains were coarse and there were more oxide inclusions near the interface when the process parameters were T = 950 °C, Ɛ = 50%, which were not conducive to the bonding properties of the stainless steel clad rebar. Therefore, no further research and analysis were carried out.

**Figure 9:**
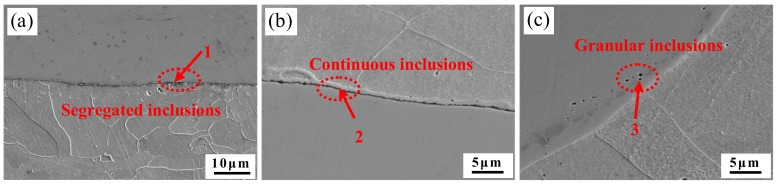
SEM images of the composite interface under different process parameters; (**a**) T = 950 °C, Ɛ = 70%; (**b**) T = 1050 °C, Ɛ = 50%; (**c**) T = 1050 °C, Ɛ = 70%.

The SEM images of the composite interface of stainless steel clad rebar for different process parameters are shown in Figure 9. It can be seen from Figure 9a that after 70% compression deformation at 950 °C, a certain number of black inclusions were formed near the interface, which were crushed under extrusion force, and finally the discontinuous aggregation state was presented near the interface. A continuous black inclusion band was present near the bonding interface in Figure 9b. This became stainless steel and carbon steel and easily formed a complex oxidation system at a higher deformation temperature of 1050 °C. When 50% compression deformation was carried out, the possibility of oxygen residue was greater, and the oxidizing elements in the steel would be oxidized. Thus, a large amount of oxidized inclusions were formed. From Figure 9c, it can be seen that the composite interface was relatively clean, and there were no aggregated or continuous inclusions, only some scattered granular inclusions of smaller size. The results showed that with increase of degree of deformation, the degree of fragmentation of interfacial inclusions was increased, and more fine and dispersed interfacial inclusions were obtained. Egea et al. [10] found that when the deformation temperature was higher, the plastic deformation force was reduced, which was beneficial to the formation of solid metallurgical bonding between stainless steel and carbon steel. The WDS analysis of the inclusions at the composite interface with different degrees of deformation is shown in Table 2.

It can be seen from Table 2, there was lower content of O in region 1 and region 3, while the content of oxygen in region 2 was greater than region 1 and region 3. This implies that with increased degree of deformation, the possibility of oxygen residue was small. In the high temperature and high oxygen environment, Si and Mn elements in region 2 were easily oxidized to form Si-Mn complex oxides, this is also stated in the relevant literature. Nomura et al. [18] found that Si-Mn oxides were easily formed on the surface of steel containing Si and Mn elements, which were considered to be sensitive to oxidation. The surface of the machined carbon steel and stainless steel has a certain roughness, and a small of O atoms will adsorb on it. These O atoms sealed at the interface preferentially react with the easily oxidized elements of Si and Mn on both sides of the interface to form a thin layer of Si-Mn oxides during long-time heating. Additionally, high content of Cr element also underwent a series of reactions under high temperature and high oxygen to form MnCr_2_O_4_ spinel oxides [19,20,21]. The Si-Mn oxides were formed in region 3 by the WDS analysis of region 3 and the analysis of region 2. In addition, MnS inclusions were also formed in region 3, the main purpose was to avoid the formation of the hot brittle phase of FeS during smelting, and a certain amount of Mn element was added to the steel, so that it reacted with S of harmful element to form MnS with high melting point, and were stable even at 1200 °C. The same phenomena and explanations were also reported in the relevant literature [22].

**Figure 10:**
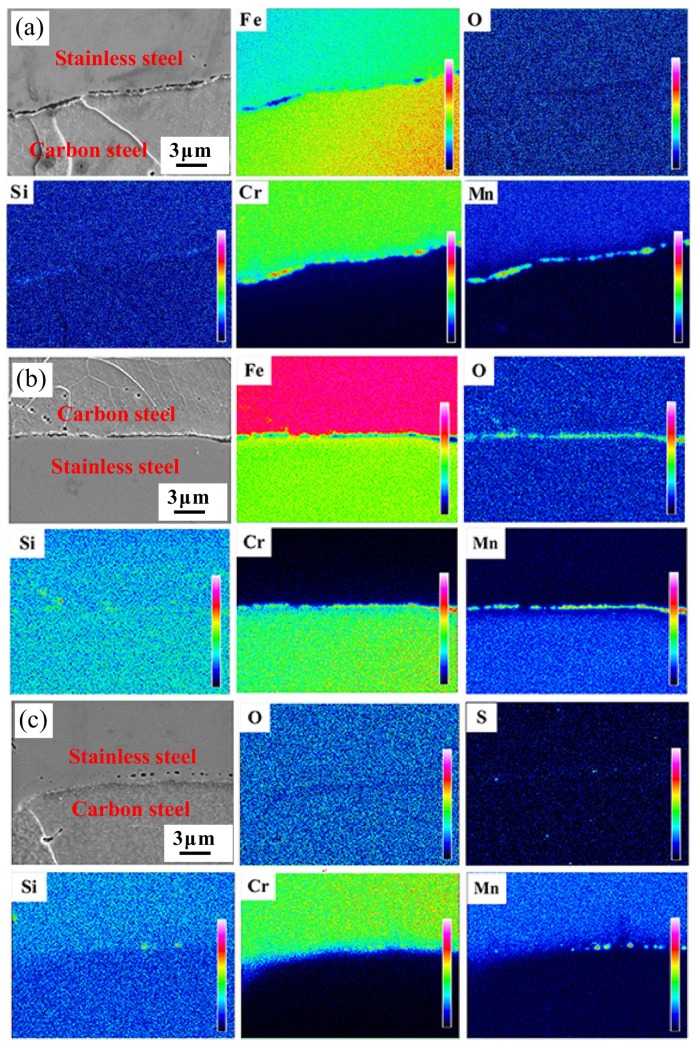
Distribution of elements at the interface for different process parameters: (**a**) T = 950 °C, Ɛ = 70%; (**b**) T = 1050 °C, Ɛ = 50%; (**c**) T = 1050 °C, Ɛ = 70%.

Based on the above analysis, the inclusions at the interface of the stainless steel clad rebar under different process parameters were considered to be Si-Mn composite oxide, MnCr_2_O_4_ oxide and MnS inclusion. In order to further determine the type of inclusions, EPMA was used to analyze the distribution of elements near the composite interface, and the results are shown in Figure 10.

It can be seen from Figure 10a that after 70% compression deformation at 950 °C, more Mn and Cr and a small amount of Si and O were enriched at the composite interface. The Si-Mn oxides were formed preferentially according to the selective oxidation mechanism, confirmed by the quantitative results of WDS analysis. While the content of O was very limited, only Si-Mn composite oxides were formed in the end. As shown in Figure 10b, a large amount of O, Si, Cr and Mn elements were segregated at the interface after 50% compression deformation at 1050 °C. The inclusions were Si-Mn composite oxides and MnCr_2_O_4_ oxides based on the quantitative results of WDS analysis and qualitative analysis of selective preferential oxidation mechanism. Similarly, the distribution of elements at the composite interface after 70% compression deformation at 1050 °C is shown in Figure 10c. The composition and type of inclusions at the interface were also determined to be Si-Mn composite oxide and MnS inclusion by quantitative analysis of WDS analysis and qualitative analysis of elements.

### 3.2. The Hardness and Bonded Performance of the Interface

**Figure 11:**
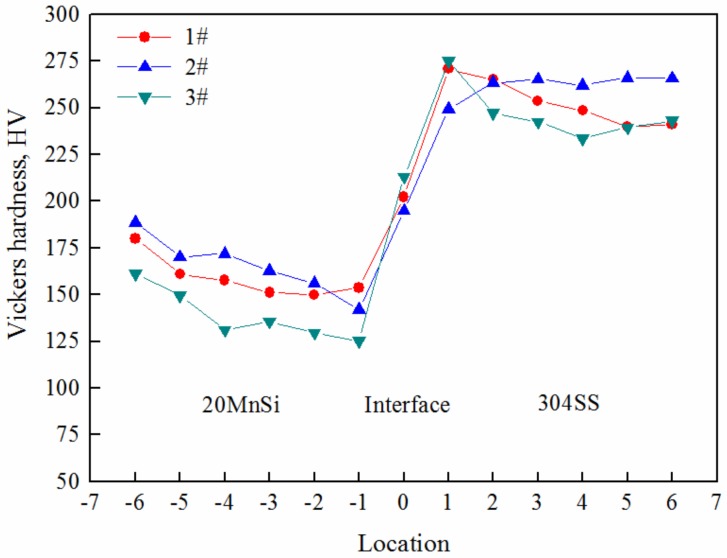
Hardness distribution near the interface under different process parameters: **1#** T = 1050 °C, Ɛ = 70%; **2#** T = 950 °C, Ɛ = 70%; **3#**T = 1050 °C, Ɛ = 50%.

In order to further study the bonded quality of the composite interface of stainless steel clad rebar, the microhardness was measured at different positions near the composite interface for different process parameters by microhardness tester. Thirteen points were taken from the stainless steel side to the carbon steel side, and the microhardness values at different locations were measured as shown in Figure 11.

It was observed from Figure 11 that closer to the bonding interface, the microhardness of stainless steel side was increased, while on the carbon steel side it was decreased slightly for different process parameters. The maximum hardness value of the bonded interface near the stainless steel side was 270 HV (Vickers Hardness), and the microhardness of carbon steel side and stainless steel side away from interface are in the range of 125–190 HV and 230–265 HV, respectively. It is known from reference [23] that element diffusion occurred in stainless steel and carbon steel during the heating process and plastic deformation. Compounds of C and Cr were formed on the stainless steel side, which increased the hardness of stainless steel side. While the carbon content near the bonded interface was decreased and the hardness also decreased due to diffusion of carbon atoms on the carbon steel side. A small number of aggregated Si-Mn oxide inclusions were formed at the interface when 70% compression deformation occurred at 950 °C. Under the process parameters, fine grains were obtained, and the number of grain boundaries increased, which hindered the movement of dislocations. After 50% deformation at 1050 °C, Si-Mn and MnCr2O4 oxides with high content were formed near the interface. All of these increased the hardness near the interface, and the grain structure far from both sides of the interface was coarsened under the process parameters. However, after 70% compression deformation, the interface was relatively clean, and only a small amount of Si-Mn oxides and MnS inclusions were dispersed. Generally, inclusions would affect the hardness. The content of pearlite increased with the increase of degree of deformation on the side of carbon steel, while the austenite grains were refined and dislocation density was gradually increased, which was favorable to the formation of fine ferrite, pearlite and bainite, and increase of hardness.

**Figure 12:**
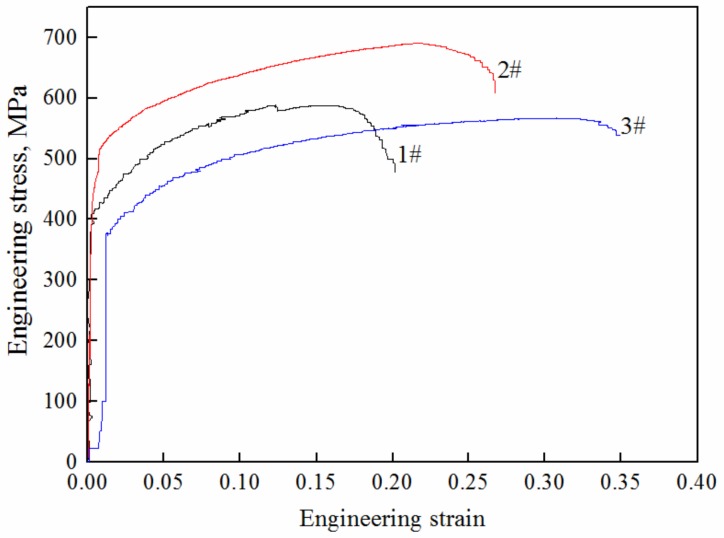
Engineering stress-strain curves of stainless steel clad rebar under different process parameters: **1**# T = 1050 °C, Ɛ = 50%; **2**# T = 1050 °C, Ɛ = 70%; **3**# T = 950 °C, Ɛ = 70%.

**Figure 13:**
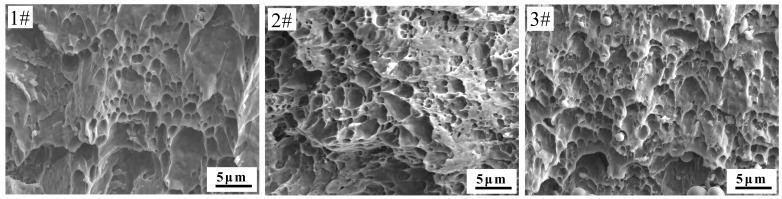
Fracture morphology of stainless steel clad rebar under different process parameters: **1**# T = 1050 °C, Ɛ = 50%; **2**# T = 1050 °C, Ɛ = 70%; **3**# T = 950 °C, Ɛ = 70%.

The engineering stress-strain curves of stainless steel clad rebar under different process parameters are shown in Figure 12. It can be seen from figure that tensile strength and elongation of stainless steel clad rebar increased when the degree of deformation was larger. When the deformation temperature was increased, the tensile strength increased, while the elongation decreased. The tensile strength was 589 MPa and the total elongation was 19% after 50% compression deformation at 1050 °C, and after 50% compression deformation, the tensile strength was 690 MPa and the total elongation was 26%, and after 70% compression deformation at 950 °C, the tensile strength was567 MPa and the total elongation was 34%. The fracture morphology of different processes was analyzed, and the results showed that all samples exhibited ductile fracture characteristics as shown in Figure 13, which indicated that the stainless steel clad rebar had good ductility.

It can be seen that under the conditions of T = 1050 °C and Ɛ = 70%, the comprehensive mechanical properties of the stainless steel clad rebar were better. Given that stainless steel and carbon steel are prone to dynamic recrystallization at a high temperature of 1050 °C, grain growth occurs due to the sufficient deformation storage energy. Moreover, a certain amount of tough phase bainite was promoted, and bainite had higher strength and higher elongation than ferrite and pearlite. When 70% of the compression deformation was carried out, the inclusions formed at the composite interface were crushed, which was conducive to the formation of solid metallurgical bond. Large compression deformation resulted in grain refinement, such that the number of grain boundaries were also increased. Due to the existence of grain boundaries, the dislocations in the deformed grains were blocked at the grain boundary, and the slip band in each grain also terminated near the grain boundary [24]. Meanwhile, there were orientation differences among the grains. In order to coordinate the deformation, it was required that each grain must experience multi-slip, and the mutual intersection of dislocations must occur when there was multiple slippage, which significantly increased the strength of the material.

At the same time, according to the engineering stress-strain curve shown in Figure 12, it can be seen that the stainless steel clad rebar did not exhibit the yield oscillation stage similar to that of single metal during tensile straining, but directly entered the strengthening stage. This was mainly due to the inconsistency of the yield limit of the two metals. When the carbon steel reached the elastic limit, the stainless steel was still in the elastic stage, and when the stainless steel reached the elastic limit, the carbon steel was already in the strengthening stage, which weakened the oscillation phenomenon in the yield stage. In addition, the curve was relatively smooth and there was no big fluctuation, which indicated that the bimetallic deformation was uniform and coordinated during the tensile process [8].

## 4. Conclusions

1. The stainless steel/carbon steel clad rebar was prepared by the thermal simulation experiment. Different structural characteristics were presented near the bonding interface, especially the distribution of inclusions after deformation at different temperatures and degrees of deformation. When T = 1050 °C and Ɛ = 70%, only a small amount of Si-Mn composite oxide and MnS inclusions were dispersed at the composite interface, and the bonding of bimetallic interface was better.
2. After 70% compression deformation at 1050 °C, three-phase structures of ferrite, pearlite and bainite were obtained in the carbon steel side, which is beneficial for the improvement of mechanical properties. The tensile test results showed that the tensile strength and the total elongation were 690 MPa and 26% respectively. A large number of dimples were distributed on the fracture surface, indicated that ductile fracture was occurred. The comprehensive mechanical properties were better than that the process parameters of T = 1050 °C, Ɛ = 50% and T = 950 °C, Ɛ = 70%.
3. The technology of thermal simulation experiment can effectively simulate the process path of a hot-rolled clad rebar, and a stainless steel/carbon steel clad rebar with excellent performance can be obtained by controlling different process conditions which can provide a theoretical basis and technical guidance for the actual hot-rolled clad rebar.

## Figures and Tables

**Table 1:** Chemical composition of clad rebar materials (mass fraction %).

Element	C	Si	Mn	P	S	Al	Cr	Ni	Fe
20MnSi	0.25	0.80	1.60	0.04	0.03	0.015	0.03	0.03	Bal.
304	0.05	1.00	2.00	0.022	0.008	0.005	19.1	8.5	Bal.

**Table 2:** Wavelength dispersive spectroscopy (WDS) analysis of the three points identified in Figure 9a–c.

Position	1	2	3
O/wt.%	1.06	12.53	1.46
Si/wt.%	0.13	4.37	0.69
S/wt.%	-	-	0.28
Cr/wt.%	6.45	15.74	9.08
Mn/wt.%	0.37	5.15	2.46
Fe/wt.%	91.06	59.12	83.06
Ni/wt.%	0.94	3.08	2.79
